# Empagliflozin mitigates endothelial inflammation and attenuates endoplasmic reticulum stress signaling caused by sustained glycocalyx disruption

**DOI:** 10.1038/s41598-022-16763-6

**Published:** 2022-07-25

**Authors:** Marc-Antoine Campeau, Richard L. Leask

**Affiliations:** 1 Department of Chemical Engineering, McGill University, Montreal, QC Canada; 2 McGill University Health Centre, Montreal, QC Canada

**Keywords:** Stress signalling, Mechanisms of disease, Endoplasmic reticulum, Mechanotransduction, Vascular diseases

## Abstract

The disruption of the endothelial cell (EC) glycocalyx (GCX) leads to cellular dysfunction promoting inflammation and cardiovascular disease progression. Recent studies have shown that empagliflozin (EMPA; Jardiance), a sodium-glucose cotransporter 2 inhibitor used in the treatment of type 2 diabetes, can improve EC functions impacted by GCX disruption although the exact cellular mechanisms remain to be elucidated. In this study, the effect of EMPA on EC inflammatory response induced by sustained GCX disruption was investigated. Human aortic ECs were cultured under shear (10 dyne/cm^2^) for 24 h with or without sustained degradation of heparan sulfate (HS). HS degradation increased inflammatory cell adhesion to ECs. EMPA (50 μM) normalized adhesion levels under sustained HS degradation. Protein expressions of eNOS, phospho-eNOS Ser1177 and ICAM-1 remained unchanged between conditions. Transcriptome analysis revealed the induction of the unfolded protein response (UPR) through the increased expression of *ATF3*, *ATF4*, *DDIT3* (CHOP), *EIF2AK3* (PERK), *HSPA5* (Grp78), *PPP1R15A* (GADD34) and *TRIB3* which was in part downregulated by EMPA. mRNA and protein expression of thioredoxin interacting protein (TXNIP) was also downregulated by EMPA. Mitigation of oxidative stress with *N*-Acetyl-l-cysteine resulted in similar reduction in inflammatory cell adhesion compared to EMPA which could indicate a potential mechanism by which EMPA normalized the inflammatory response. In conclusion, this study demonstrated the potential of EMPA to resolve the inflammatory response of ECs caused by sustained GCX disruption while altering UPR signaling under endoplasmic reticulum stress.

## Introduction

The endothelial glycocalyx (GCX) is a glycosaminoglycan and proteoglycan-rich functional layer lining the endothelium which promotes homeostasis through its role as a mechanosensor and as a barrier to inflammatory cell attachment. Disruption of the GCX in pathological conditions such as type 2 diabetes (T2D) and atherosclerosis^1,2^ causes endothelial cell (EC) dysfunction and thus, leads to cardiovascular disease progression. In particular, in vitro and in vivo degradation of heparan sulfate (HS), the main component of EC GCX, has been shown to impair shear-regulated nitric oxide (NO) production and promote adhesion of inflammatory cells^3–5^. Given its central role in EC homeostasis, the GCX is considered a potential therapeutic target to prevent or alleviate EC dysfunction and therefore benefit clinical outcomes^6,7^.

Empagliflozin (EMPA; Jardiance ^®^) belongs to the sodium-glucose cotransporter 2 inhibitor (SGLT2i) class of drugs used in the treatment of T2D and more recently heart failure. This class has emerged as a promising new therapy for cardiovascular diseases as treatment with a SGLT2i has shown impressive beneficial outcomes in recent clinical trials (e.g. EMPA-REG OUTCOME (NCT01131676)^8^, DAPA-HF (NCT03036124) and EMPEROR-Reduced (NCT03057977)^9,10^). Among these, the EMPA-REG OUTCOME trial demonstrated significant reductions in the rate of hospitalization for heart failure and cardiovascular death in T2D patients treated with EMPA when compared with placebo^8^. Subsequent animal studies have suggested pleiotropy and extended protective effects by EMPA to atherosclerosis^11^, heart failure^12,13^ and ischemia–reperfusion injury^14^ in non-diabetic conditions, suggesting that the cardiovascular protection is not achieved solely through glycosuria.

As endothelial dysfunction is a central step in the development of cardiovascular complications, it has been hypothesized that SGLT2i can prevent or overcome EC dysfunction. Treatment with EMPA has been shown to improve EC-dependent vasorelaxation in diabetic rodents^15–17^. El-Daly et al. showed similar results with ex vivo mice aortic tissue in response to hyperglycemic conditions^18^. Moreover, EMPA reduced the inflammatory response and the adverse vascular remodeling in Ang II-induced AAA of ApoE^(−/−)^ mice. Adhesion of inflammatory cells and adhesion molecule expressions in isolated mouse ECs were also reduced^19^. Recent in vitro studies suggested that EMPA restored NO bioavailability and reduced oxidative stress in TNF-α treated ECs^20,21^. Although cardiac and vascular protective mechanisms have been proposed^22^, the exact mechanism by which EMPA promotes EC homeostasis and in turn contributes to better vascular health remains to be clarified.

Considering the importance of GCX health in EC function and the impressive ability of EMPA to improve cardiovascular outcomes, we hypothesized that EMPA can overcome EC dysfunction caused by GCX disruption which could, in part, explain the improvement in vascular health. We previously showed that EMPA can normalize the inflammatory response of ECs possibly through shear mechanotransduction of a restored GCX after acute degradation^23^. As the GCX is a dynamic surface layer with potential of regrowth, this work aims at exploring more closely the signaling pathways involved under sustained GCX disruption to identify other anti-inflammatory mechanisms of EMPA.

## Results

### EMPA induces an anti-inflammatory response independent of HS

**Figure 1:**
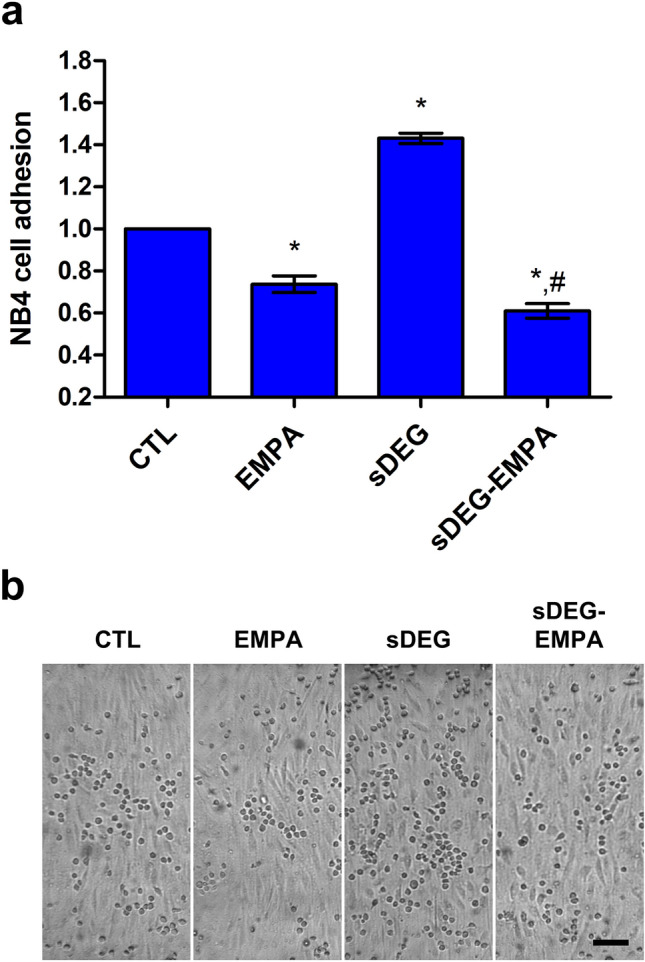
NB4 cell adhesion assay following shear culture for 24 h. (**a**) Relative numbers of NB4 cell adhesions to TNF-α stimulated ECs under sustained HS degradation with or without EMPA treatment. Degradation caused increases in adhesion compared to control (sDEG vs CTL *p < 0.001). EMPA reduced the adhesion compared to control (EMPA vs CTL *p < 0.001) and prevented the increase caused by degradation (sDEG-EMPA vs sDEG ^#^p < 0.001) resulting in adhesion levels below control (sDEG-EMPA vs CTL *p < 0.001). (**b**) Representative images of each condition (scale bar = 100 μm).

Sustained HS degradation was used to evaluate the inflammatory response of ECs to EMPA independent of HS. Inflammatory cell adhesion assays were performed on TNF-α treated cells similar to previous studies^3,23,24^. EMPA significantly reduced NB4 cell adhesions compared to control (Fig. 1, EMPA vs CTL *p < 0.001). While sustained degradation caused significant increases in NB4 cell adhesion (sDEG vs CTL *p < 0.001) showing an enhanced inflammatory response, EMPA reduced the adhesions to the same level as non-degraded samples (sDEG-EMPA vs sDEG ^#^p < 0.001) and below control (sDEG-EMPA vs CTL *p < 0.001). This suggests that EMPA prevents the inflammatory response of ECs to TNF-α stimulation and HS degradation. As it persists under sustained degradation, it can be assumed that EMPA can reduce inflammation independently of HS.

### ICAM-1 and eNOS are not implicated in the HS independent EMPA anti-inflammatory response

**Figure 2:**
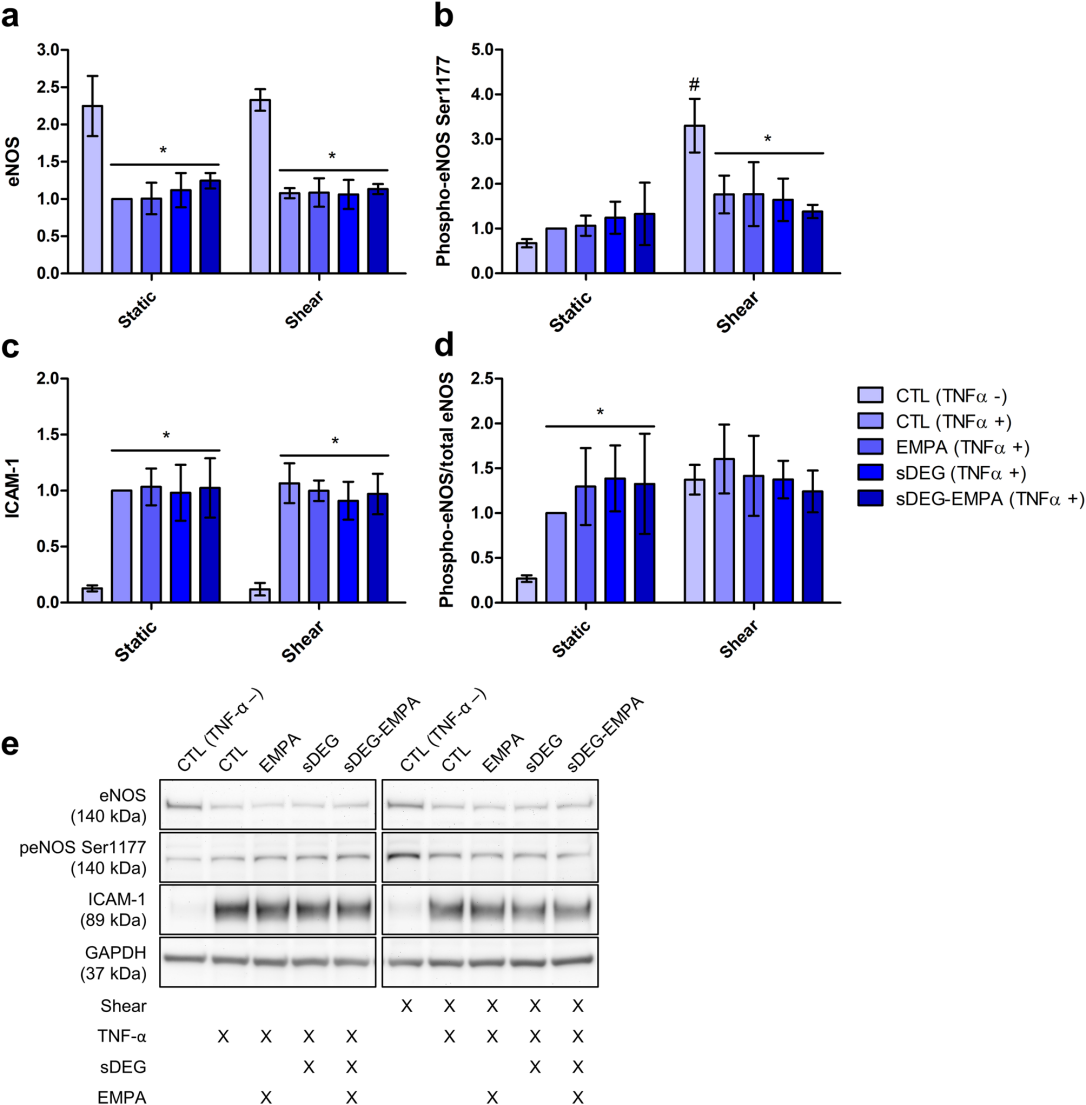
Protein expression quantification through western blot densitometry measurements after 24 h of culture. Expression of (**a**) eNOS, (**b**) phospho-eNOS Ser1177, (**c**) ICAM-1 and (**d**) the ratio phospho-eNOS/total eNOS were assessed. Shear increased the expression of phospho-eNOS Ser1177 compared to the static control (CTL TNF-α – shear vs CTL TNF-α – static ^#^p < 0.001). TNF-α stimulation reduced expression levels of eNOS in static and shear culture and reduced phospho-eNOS Ser1177 in shear culture compared to TNF-α – controls (*p < 0.001). ICAM-1 expression was upregulated by TNF-α in static and shear culture compared to TNF-α – controls (*p < 0.001). Phospho-eNOS/total eNOS ratios were increased in static condition compared to the TNF-α – control (*p < 0.01) while no significant differences were observed in shear condition. No significant effects of degradation and EMPA were observed on the protein expression levels within TNF-α groups. (**e**) Representative bands and culture condition details. Full-length blots can be found in the Supplementary Fig. S4.

The expression of eNOS and ICAM-1 were assessed to characterize the inflammatory response induced by sustained HS degradation and their potential role in the recovery effect provided by EMPA. After 24 h of culture, eNOS expression in all static and shear TNF-α + conditions were reduced compared to TNF-α – controls (Fig. 2a, *p < 0.001). There was no observed effect of EMPA nor degradation on eNOS expression. Shear significantly increased the phospho-eNOS Ser1177 levels compared to the static control in absence of TNF-α (Fig. 2b, CTL TNF-α – shear vs CTL TNF-α – static ^#^p < 0.001). TNF-α stimulation impaired the shear-induced increase of phospho-eNOS Ser1177 to similar levels in all shear conditions suggesting reduced eNOS activation (*p < 0.001). Again, no significant impact on phospho-eNOS Ser1177 caused by EMPA or degradation were found. Similarly, TNF-α induced the expression of ICAM-1 to similar levels in all conditions (Fig. 2c, *p < 0.001) with no significant effect of shear, EMPA or degradation. Interestingly, the phospho-eNOS Ser1177/total eNOS ratio remained unchanged with or without TNF-α in shear condition while significantly higher ratios were found in static culture (Fig. 2d, *p < 0.01). Taken together, these results suggest that the activation of eNOS and the expression of ICAM-1 are not directly implicated in the HS independent recovery of EC dysfunction by EMPA.

### The induction of the unfolded protein response caused by sustained HS degradation is partially restored by EMPA

To explore the possible signaling pathways impacted by the sustained degradation of HS and EMPA beyond the traditional mechanobiology and anti-inflammatory role of the GCX, a genome-wide transcriptome analysis was performed. We sought to better understand the mechanisms by which EMPA prevents the increased inflammatory response triggered by the sustained HS degradation under shear culture. EC dysfunction associated genes were screened and grouped to identify the signaling pathways involved based on their fold changes.

Shear exposure for 24 h showed regulation of hallmark genes such as eNOS (*NOS3*), KLF2 and COX-2 (*PTGS2*) while downregulating inflammatory signaling genes (*IL1B*, *IL6*) compared to the static control (CTL vs CTL (Static)). mRNA expression of ICAM-1 was also slightly downregulated by shear (Supplementary Table S1).

**Figure 3:**
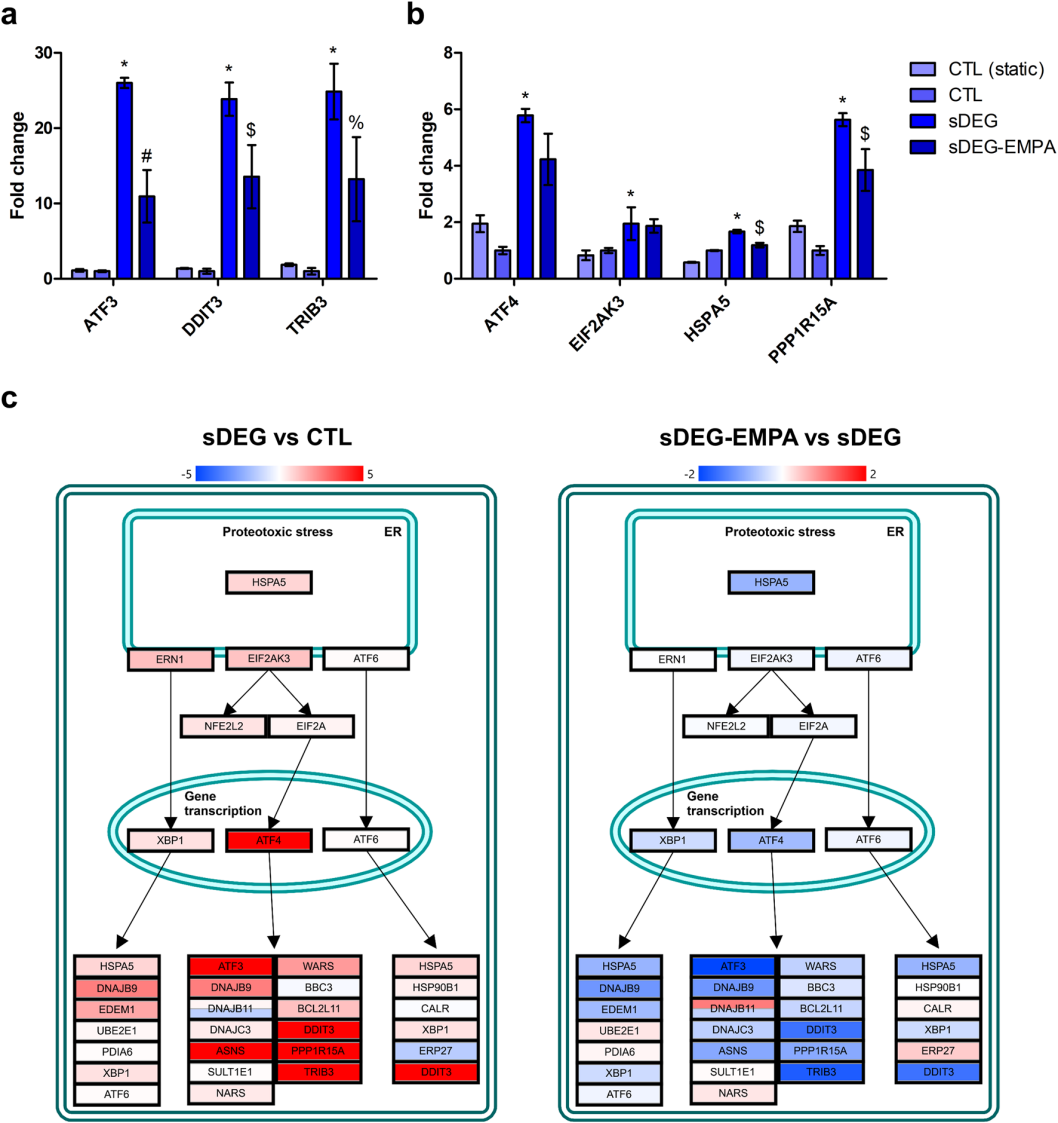
Impact of sustained HS degradation and EMPA treatment on ER stress gene transcription. (**a**) Fold changes of *ATF3*, *DDIT3* (CHOP), *TRIB3* and (**b**) *ATF4*, *EIF2AK3* (PERK), *HSPA5* (GRP78) and *PPP1R15A* (GADD34). Sustained degradation caused significant fold increases in *ATF3* (26.01), *ATF4* (5.78), *DDIT3* (23.85), *EIF2AK3* (1.95), *HSPA5* (1.67), *TRIB3* (24.86) and *PPP1R15A* (5.63) expression (sDEG vs CTL *p < 0.001). EMPA significantly reduced the expression of *ATF3* (− 2.37), *DDIT3* (− 1.76), *HSPA5* (− 1.40), *PPP1R15A* (− 1.46) and *TRIB3* (− 1.88) (sDEG-EMPA vs sDEG ^#^p < 0.001, ^$^p < 0.01, ^%^p < 0.05). (**c**) Simplified ER stress signaling pathway generated from the Transcriptome Analysis Console software showing regulation through the PERK/eIF2/ATF4 branch of the UPR. The original version of the pathway is made available through WikiPathways^25^.

Sustained HS degradation under shear caused the upregulation of genes related to the unfolded protein response (UPR) and the C/EBP homologous protein (CHOP) transcriptional factors suggesting that ECs were subjected to endoplasmic reticulum (ER) stress. As such, activating transcription factor 3 (*ATF3*), DNA-damage-inducible transcript 3 (*DDIT3*; CHOP) and tribbles homolog 3 (*TRIB3*) were significantly upregulated under HS degradation compared to the control (Fig. 3a, sDEG vs CTL *p < 0.001). The activation of the UPR was also apparent by the upregulation of the activating transcription factor 4 (*ATF4*), the eukaryotic translation initiation factor 2 α kinase 3 (*EIF2AK3*; PERK), the ER chaperon protein Grp78 (*HSPA5*) and the growth arrest and DNA damage-inducible protein GADD34 (*PPP1R15A*) (Fig. 3b, sDEG vs CTL *p < 0.001). EMPA significantly reduced the expression of *ATF3*, *DDIT3*, *HSPA5, PPP1R15A* and *TRIB3* under HS degradation (sDEG-EMPA vs sDEG ^#^p < 0.001, ^$^p < 0.01, ^%^p < 0.05). The transcriptional regulation induced by sustained HS degradation and EMPA appeared to be mediated mainly through the PKR-like ER kinase (PERK, *EIF2AK3*)/eukaryotic initiation factor 2 (eIF2)/ATF4 branch of the UPR (Fig. 3c). Taken together, these results suggest that HS degradation under shear promotes EC dysfunction through the activation of the UPR and in turn, CHOP-mediated apoptosis, features of unresolved ER stress. Treatment with EMPA showed attenuation of ER stress through the transcriptional downregulation of key UPR genes.

### EMPA potentially attenuates inflammation through the regulation of TXNIP

**Figure 4:**
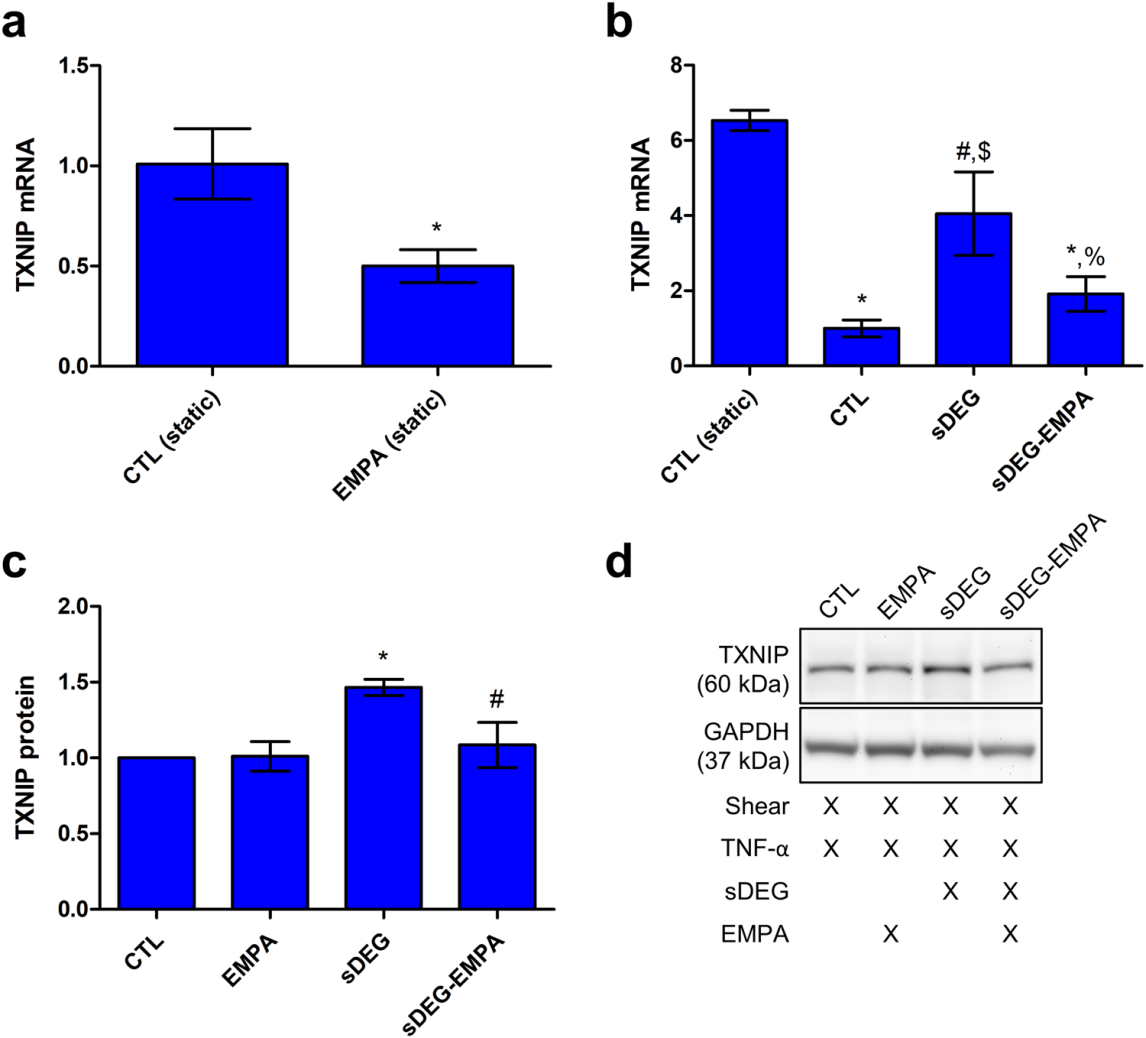
TXNIP mRNA expression from transcriptome analysis and protein expression in static and shear culture. (**a**) EMPA significantly reduced TXNIP mRNA levels after 6 h of static culture (EMPA vs CTL *p < 0.05) (conditions in triplicates). (**b**) TXNIP mRNA level is differently regulated by shear, sustained HS degradation and EMPA after 24 h. Shear downregulated TXNIP mRNA compared to the static control (CTL vs CTL (Static) *p < 0.001) while sustained degradation partly abolished shear-induced downregulation (sDEG vs CTL ^#^p < 0.01, sDEG vs CTL (Static) ^$^p < 0.01) in TNF-α stimulated ECs. EMPA treatment restored the shear-induced downregulation compared to sDEG (sDEG-EMPA vs sDEG ^%^p < 0.05, sDEG-EMPA vs CTL (Static) *p < 0.001) (conditions in triplicates). (**c**) TXNIP protein expression levels in shear culture are increased under degradation compared to the control (sDEG vs CTL *p < 0.01) in TNF-α stimulated ECs. EMPA reduced the expression under degradation (sDEG-EMPA vs sDEG ^#^p < 0.01), normalizing the expression compared to the control. (**d**) Representative bands and culture condition details. Full-length blots can be found in the Supplementary Fig. S4.

In parallel to the UPR, EMPA was found to reduce the expression of the thioredoxin interacting protein (TXNIP) which impacts oxidative stress regulation and has been related to ER stress. Treatment with EMPA for 6 h in static culture was found to downregulate the mRNA expression of TXNIP (Fig. 4a, EMPA vs CTL *p < 0.05). TXNIP mRNA levels were also significantly reduced by shear after 24 h (Fig. 4b, CTL vs CTL (Static) *p < 0.001). Sustained HS degradation partially abolished the shear-induced regulation (sDEG vs CTL ^#^p < 0.01, sDEG vs CTL (Static) ^$^p < 0.01) and treatment with EMPA normalized the expression by significantly reducing the increased levels caused by degradation (sDEG-EMPA vs sDEG ^%^p < 0.05, sDEG-EMPA vs CTL (Static) *p < 0.001). Sustained HS degradation also caused a significant increase in TXNIP protein expression (Fig. 4c, sDEG vs CTL *p < 0.001) and similar to the mRNA levels, EMPA normalized the expression compared to the control (sDEG-EMPA vs sDEG ^#^p < 0.01). This suggests that TXNIP could be a potential mediator in the ER stress response as its mRNA and protein expression was modulated by shear, sustained HS degradation and EMPA.

### *N*-Acetyl-l-cysteine treatment reduces NB4 cell adhesion similar to EMPA

**Figure 5:**
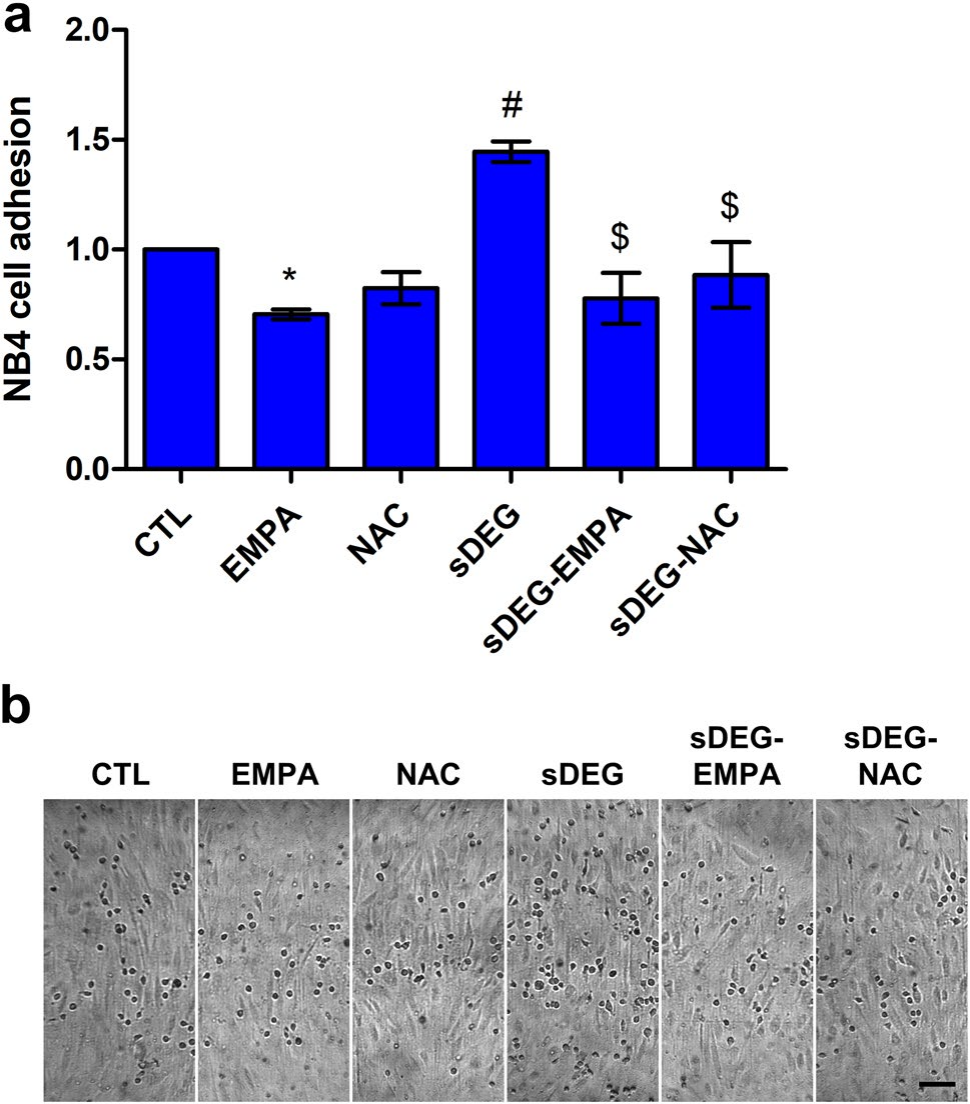
NB4 cell adhesion assay with NAC to mitigate oxidative stress. (**a**) Relative numbers of NB4 cell adhesions to TNF-α stimulated ECs following treatments and sustained HS degradation under shear culture. EMPA reduced adhesion compared to control (EMPA vs CTL *p < 0.05). Degradation caused increases in adhesion compared to control (sDEG vs CTL ^#^p < 0.001). Treatment with EMPA or NAC reduced the adhesion under degradation (sDEG-EMPA and sDEG-NAC vs sDEG ^$^p < 0.001) and restored normal adhesion level. (**b**) Representative images of each condition (scale bar = 100 μm).

To further study the reduction in NB4 cell adhesion caused by EMPA, we sought to determine if the effect was mediated through the regulation of oxidative stress under shear and sustained HS degradation. Prior to the adhesion assay, ECs were treated with N-Acetyl-l-cysteine (NAC) during shear exposure for 24 h. Unlike EMPA, NAC did not significantly reduce the number of adhered NB4 cells compared to the control (Fig. 5, NAC vs CTL). However, under sustained degradation, treatment with NAC resulted in a significant reduction in adhesions (sDEG-NAC vs sDEG ^$^p < 0.001), comparable to the effect provided by EMPA (sDEG-EMPA vs sDEG ^$^p < 0.001). This could suggest that the mitigation of oxidative stress during shear exposure is a potential mechanism by which EMPA-treated ECs reduce their inflammatory response to sustained HS degradation.

## Discussion

The disruption of the endothelial GCX has been associated with pathological conditions of the vasculature and impaired endothelial function making the role of a healthy GCX of interest in multiple diseases and treatment options. Normal GCX mechanotransduction helps maintain EC homeostasis through shear-regulated functions such as NO-driven vasodilation, cell barrier function and inflammatory cell transmigration. In this regard, treatment with SGLT2i has been demonstrated to improve vascular functions in vivo and in vitro in non-hyperglycemic conditions through improvement of EC homeostasis^13,17,19–21,26,27^. Recent studies have demonstrated that EMPA can improve EC function but the mechanisms involved remain elusive.

Given the potential of EMPA to remediate EC dysfunction, we sought to study the effects of EMPA in a pro-inflammatory chronic GCX disruption model by using sustained HS degradation. In this study, we were able to show the impact of sustained GCX disruption on the induction of ER stress and a possible mechanistic effect of EMPA to attenuate the associated inflammatory response which could contribute to the improved vascular health observed clinically^8–10^.

Under normal conditions, the GCX at the apical surface of ECs allows the transduction of shear stress through the cytoplasmic membrane, transforming sensing forces into biochemical signals^28^. Thus, laminar shear stress induces the adaptive remodeling of the cytoskeleton i.e. cell elongation and the production of NO through the activation (phosphorylation Ser1177) of eNOS^29,30^. Upon disruption of the surface GCX, these transduced effects are impaired, leading ECs to a pro-inflammatory state, promoting inflammatory cell adhesion^3^. Hyperglycemic conditions have been shown to cause vascular GCX disruption in vivo^1,31^ and in vitro^32^ thus treatment with EMPA was studied for its potential benefits on HS integrity, which could prevent EC dysfunction.

The sustained HS degradation in our study reduced the HS mean intensity at EC surface by approximatively 85% and prevented regrowth during the 24 h culture period (Supplementary Fig. S1) which is in line with previous in vitro studies^33,34^. EMPA did not improve HS intensity in control condition or under sustained degradation. Similarly, treatment with EMPA did not overcome the shear-induced elongation loss under sustained HS degradation (Supplementary Fig. S2) but resulted in significantly less NB4 cells adhered to the EC surface under degraded condition (Fig. 1). Taken together, these results define the mechanosensory and anti-inflammatory role of the GCX as HS-dependent and indicate that EMPA can reduce inflammation independently of HS-mediated EC functions.

The impact on inflammation downstream of known GCX mechanotransduction was assessed through eNOS homeostasis and ICAM-1 expression to characterize the inflammatory response of treated ECs. Degradation of HS has been linked to reduced shear-induced NO production in ECs^5,35^. Ebong et al. and others further demonstrated that the impairment of NO production is mediated through reduced phospho-eNOS Ser1177 levels and is concomitant with loss of EC remodeling^30,36^. In our case, stimulation with TNF-α resulted in significant reductions in eNOS and phospho-eNOS Ser1177 levels with no further decrease due to HS degradation, indicating that TNF-α may have eclipsed the impact of degradation on NO production (Fig. 2). Nonetheless, no improvement of phospho-eNOS/eNOS levels were found with EMPA, indicating that eNOS activation might not contribute directly to the anti-inflammatory effect of EMPA. Although we did not find significant changes in the expression level of phospho-eNOS/eNOS induced by EMPA, recent studies have shown improved NO production and EC-dependent vasodilation in response to EMPA. Treatment with 1 μM EMPA restored NO bioavailability in TNF-α stimulated cardiac microvascular ECs (CMECs)^21^ and human coronary arterial ECs (HCAECs) while eNOS phosphorylation levels remained unchanged under static conditions^20^. Conflicting results exist regarding the effects of EMPA on inflammation and adhesion molecule expressions. Ortega et al. showed the reduction in ICAM-1 and VCAM-1 expression in aortic sections of Ang-II stimulated mice^19^. Moreover, reduction of inflammatory cell adhesion in a dose-dependent manner was demonstrated with Ang-II stimulated HUVECs. Expression of ICAM-1 and VCAM-1 was also found to be reduced in CMECs isolated from EMPA-treated mice^17^. Conversely, expression of ICAM-1 and VCAM-1 remained unchanged in TNF-α stimulated CMECs^21^ and HCAECs^20^ treated with 1 μM of EMPA. We previously showed that ICAM-1 expression correlates with NB4 cell adhesion^3^, however, the protein expression of ICAM-1 (Fig. 2) were unaffected by sustained HS degradation or EMPA suggesting that regulation of the adhesion molecule ICAM-1 may not contribute to the variations in the number of NB4 cell adhesions.

From the transcriptome analysis, genes form the PERK/eIF2/ATF4 branch of the UPR were identified as being significantly regulated by sustained HS degradation and EMPA, indicative of ER stress induction (Fig. 3). Sustained degradation caused the concomitant upregulation of *ATF3* and CHOP (*DDIT3*), likely through the signaling cascade of eIF2α phosphorylation and ATF4 transcriptional regulation^37^. Downstream of CHOP, upregulation of GADD34 (*PPP1R15A*) and TRIB3 are related to the inhibitory regulation of eIF2α phosphorylation and ER stress-mediated cell apoptosis^38^, respectively. Together, these changes evoke the progressive induction of UPR due to unresolved ER stress leading to increase reactive oxygen species (ROS) production and the activation of apoptotic signaling through CHOP^39–41^. To the best of our knowledge, very few studies have established a direct link between GCX degradation and ER stress. Recently, Dhounchak et al. demonstrated the correlation between the loss HS proteoglycans and the upregulation of ER stress gene expression (*DDIT3* (CHOP), *HSPA5* (Grp78) and *ATF3*) in beta cells of diabetic murine models^42^. Similar observations were made with MIN6 cells where the induction of ER stress with thapsigargin caused the degradation of intracellular HSPG and HS, suggesting a degradation mechanism at the cellular level. Moreover, shear is known to have a regulatory effect on ER stress. While laminar shear stress limits ER stress^43^, low shear or disturbed flow promote ER stress signaling with inflammation and apoptosis^44,45^. This could, in part, explain how sustained HS degradation triggers ER stress through the loss of normal laminar shear stress response.

Treatment with EMPA caused small but significant reductions in the transcriptional expression of *ATF3*, *DDIT3* (CHOP), *HSPA5* (Grp78), *PPP1R15A*, and *TRIB3* under HS degradation suggesting a dampened apoptotic response (Fig. 3). Other studies have also found similar dampening effects of EMPA on ER stress. Treatment of ApoE^(−/−)^ and C57Bl/6 mice with EMPA for 5 weeks resulted in the reduction of hepatic ER stress as shown by the downregulation of ER stress signaling genes including *HSPA5* (Grp78), *ERN1* (IRE1), *XBP1*, *DDIT3* (CHOP), *ATF4*, *ATF6* and *GADD45A* and the reduced ratio of phospho-eIF2α/eIF2α^46,47^. A study by Zhou and Wu demonstrated the potency of EMPA on ER stress related cardiomyopathy of diabetic rats^48^. Treatment with EMPA for 8 weeks resulted in improved cardiac functions along with reduced cardiomyocyte apoptosis and protein expression of ER stress markers (Grp78, CHOP and cleaved caspase-12) suggesting a lesser impact of ER stress on the cardiomyocytes.

ER stress is inherently tied to oxidative stress as the adaptive protein folding is coupled to ROS production^49^. Additionally, oxidative stress can be exacerbated through the action of TXNIP which is related to ER stress by its concomitant activation. TXNIP is a critical regulator of ROS signaling implicated in diverse vascular pathologies due to its role in downstream inflammation and apoptosis. Through its inhibitory effect, TXNIP can impair the thiol-redox system thioredoxin (TRX), promoting oxidative stress. Lerner et al. demonstrated how activation of the UPR sensors, IRE1α and PERK, resulted in increased TXNIP mRNA levels and increased ROS levels in mouse embryonic fibroblasts^50^. Deletion of IRE1α, PERK or TXNIP abolished the detrimental effects associated with unresolved ER stress. Moreover, disturbed flow has been shown to upregulate TXNIP protein expression which caused increased leukocyte–EC adhesion through increased ICAM-1 and VCAM-1 expression^51^. TXNIP is also known to interact with and activate the NLRP3 inflammasome resulting in enhanced inflammation as well as promoting apoptosis through the dissociation of TRX from the apoptosis signal-regulating kinase 1 (ASK1)^52,53^.

Given the detrimental effects associated to the expression of TXNIP and its implication in ER stress, we hypothesize that it plays a regulatory role in the effects provided by EMPA. Indeed, TXNIP mRNA levels were downregulated by EMPA after 6 h in static condition (Fig. 4). Sustained HS degradation caused the loss of the shear-induced downregulation of TXNIP mRNA and an increase in TXNIP protein. EMPA respectively reduced and normalized the mRNA and protein expression, which correlates with the trend observed in NB4 cell adhesion. These results are in accordance with previous studies showing the role of shear-regulated TXNIP in the inflammatory response of HUVECs^52^ and the correlation between TXNIP expression and leukocyte–endothelium adhesiveness^51^.

The transcriptomic profile of degraded samples (sDEG and sDEG-EMPA vs CTL) was also characterized by the differential regulation of redox genes (Supplementary Table S1) suggesting an adaptive response to oxidative stress. While we were unable to show the direct reduction of ROS by EMPA in static culture (Supplementary Fig. S3) or under shear (data not shown), a few studies have shown that SGLT2i can effectively reduce oxidative stress in different conditions. Pre-clinical studies demonstrated the potential of EMPA to reduce whole blood oxidative burst, cardiac NAPDH oxidase activity as well as aortic and endothelial ROS levels in diabetic rats^15,16^. Zhou et al. also showed reduction in intracellular and mitochondrial ROS by EMPA in CMECs isolated from diabetic rats^17^. In vitro treatment of CMECs/HCAECs with 1 μM EMPA resulted in the reduction of TNF-α induced oxidative stress as measured by intracellular and mitochondrial ROS suggesting that EMPA has a protective effect against ROS production in normoglycemic conditions while a direct anti-oxidant effect was excluded by the authors.

By virtue of its role in EC homeostasis, the disruption of the GCX also coincide with increased ROS production, promoting a vicious circle of degradation as ROS will further promote GCX disruption^54,55^. In this context, unresolved ER stress with increased TXNIP expression can cause ROS production and may result in increased NB4 cell adhesion. To test this, NAC was used to promote ROS scavenger and mitigate oxidative stress during shear exposure. Perfusion of NAC has been previously shown to prevent hyperglycemic damage of the glycocalyx^1^ and to reduce the level of hyaluronan shedding during renal fluid resuscitation^56^. In our case, incubation with NAC resulted in similar reductions of adhesion compared to EMPA suggesting an oxidative stress mediated mechanism by EMPA. However, the exact impact of EMPA on the interplay between ER stress and TXNIP, resulting in reduced oxidative stress and in turn reduced inflammation, remains to be clarified. TXNIP knockout ECs could represent an interesting model to test the EMPA effect dependency on TXNIP expression.

The study of the glycocalyx in vitro as well as conclusions drawn from transcriptional data comes with inherent limitations. First, the glycocalyx of ECs in culture is known to lack the thickness of its in vivo counterpart which may lessen the impact of degradation on inflammatory cell adhesion^57^. Although different from chronic GCX disruption, sustained enzymatic degradation of HS was used as an inflammatory stimulus to mimic EC GCX shedding as found in diabetic or curved/branching vasculature. Furthermore, while the effect of HS degradation was the focus of this study, degradation of other components of the GCX can also impact EC homeostasis which could induce different signaling cascades and therefore change the role of EMPA in the restoration of GCX functions. Although the use of TNF-α under sustained HS degradation does not allow to fully dissociate their respective impact on EC inflammation in all experiments, TNF-α addition was required to perform adhesion assays and was therefore use in subsequent experiments to properly interpret the results. The single time-point studied does not reflect transient response or feedback regulation between ER stress and inflammation. Moreover, a suprapharmacological concentration of EMPA was used compared to the reported serological concentrations of treated patients^58^ in order to study the reduction in inflammation previously reported and to highlight the potential cellular mechanisms that may occur under lower serological concentrations over longer periods of time.

In conclusion, the present study suggests a possible mechanism by which EMPA may contribute to better endothelial health. Our results point toward the potential of EMPA to attenuate EC inflammation independently of HS-mediated functions, as shown by the reduction of inflammatory cell adhesion under sustained HS degradation. Downregulation of pro-apoptotic signaling and TXNIP expression by EMPA may contribute to oxidative stress mitigation therefore limiting oxidative damage under unresolved ER stress and resulting in reduced inflammatory cell adhesion. Future work should aim at studying the regulatory mechanisms associated with the expression of TXNIP on the inflammatory response through the NLRP3 inflammasome and apoptotic signaling. A better understanding of the effects of SGLT2i on GCX-mediated EC functions could benefit new strategies to limit EC dysfunction in pathological conditions where chronic GCX damage is found.

## Materials and methods

### Cell culture and 3D culture model

Human abdominal aortic endothelial cells (HAAECs, ATCC, Coriell, CRL-2472) were cultured in endothelial cell growth medium (Promocell, C-22020), supplemented with 10% fetal bovine serum and 1% penicillin–streptomycin (Invitrogen) in culture flasks coated with 0.1% pig gelatin at 37 °C and 5% CO_2_. Cells were grown to confluence and harvested from the flask with TrypLE (ThermoFisher, 12605028). Experiments were performed with cells at passage 5 and 6.

Tubular cell culture models were produced as previously described^59,60^. Briefly, the silicon elastomer Sylgard™ 184 (Paisley, AVDC0184-39) was prepared by mixing the base and curing agent in a 10:1 ratio. Sylgard™ 184 was cured around a polished stainless steel rod in plexiglass molds. Following complete curing, the rod was removed, creating a straight hollow tube where cell can be seeded and grown. In preparation to cell culture, models were sterilized by boiling and coated with a 40 μg/mL human fibronectin (Akron Biotech, AK9715-0005) solution overnight. HAAECs were seeded at a concentration of 1 × 10^6^ cells/mL and grown for 48 h. Models were mounted on a rotor to ensure complete and even coverage of the inner surface. Cells were then cultured for 24 h in static or shear condition (laminar shear stress of 10 dyne/cm^2^) with or without 50 μM EMPA.

### Heparan sulfate enzymatic degradation

GCX disruption was performed by enzymatic degradation of HS. HAAECs were treated with 0.5 U/mL of heparinase III (Sigma unit, enzyme 4.2.2.8, IBEX Pharmaceuticals, 50-012) in FBS-free media for 2 h followed by the addition of 0.1 U/mL in the medium during the 24 h of static or shear culture to achieve sustained degradation (sDEG) and to prevent HS regrowth. The initial condition of degradation (DEG T0) was assessed immediately after the initial 2 h exposure to heparinase III.

### Heparan sulfate immunostaining and confocal microscopy

Cells were fixed in situ with a 2%/0.1% paraformaldehyde/glutaraldehyde solution in PBS for 30 min without prior washes to prevent HS disruption. Sections of the models were cut and samples were blocked with a 2% normal goat serum (NGS) in PBS. Primary antibody for HS (1:100, mouse, 10E4, Amsbio, 370255) was diluted in 1% NGS/PBS and incubated overnight at 4 °C. Cells were rinsed with PBS and incubated for 1 h with the secondary antibody (ThermoFisher, goat anti-Mouse IgG, IgM (H + L) Alexa Fluor 488, A-10680) and DAPI (1:1000) diluted in 1% NGS/PBS. Cells were rinsed with PBS and the model section were cut, mounted using 0.2% Dabco/Glycerol (1:5, Sigma) and imaged via laser scanning confocal microscopy (Zeiss Exciter 800). Z-stack images were acquired at 10 × magnification and maximum intensity projections were created to quantify the mean gray value of each image using ImageJ.

### Cell morphology and shape index

Following the static or shear culture with or without EMPA, cells were fixed in situ with 1% paraformaldehyde in PBS for 20 min, rinsed with PBS and stained with a 4% crystal violet (BD Biosciences) solution for 10 min. Cell morphology was assessed through light microscopy (Leica DMIL microscope and Leica DC300 camera) images and quantified by computing the shape index of individual cells using Matlab scripts^59^. The shape index (SI) is used as a metric of cell elongation where a perfectly round cell has a SI close to 1 and a straight elongated cell, a SI closer to 0.

### Adhesion assay

Adhesion assays were performed as previously described^24^. Acute promyelocytic leukemia (NB4) cells were cultured in suspension and maintained at a concentration of 2 × 10^5^ to 1 × 10^6^ cells/mL in RPMI 1640 medium with 2 mM l-glutamine supplemented with 10% FBS and 1% penicillin–streptomycin. 48 h prior to the adhesion assay, NB4 cells were stimulated with 10^–6^ M all-trans-retinoic acid (ATRA, Sigma, R2625) to induce differentiation into granulocytes. Adhesion assays were conducted following 24 h of shear exposure and tumor necrosis factor-alpha (TNF-α; PeproTech, 300-01A, 10 ng/mL) stimulation of HAAECs to induce NB4 cell adhesion. As previously shown, TNF-α stimulation is necessary to reach significant numbers of NB4 cell adhesions^24^. Also, to assess the effect of oxidative stress on adhesion, HAAECs were treated with 1 mM of *N*-Acetyl-l-cysteine (NAC, Sigma, A9165) for 24 h prior to the adhesion assay. A NB4 cell suspension at 5 × 10^5^ cells/mL was perfused through the models at a shear stress of 1.25 dyne/cm^2^ for 1 h. PFA 1% was then perfused to fix the adhered NB4 cells to the endothelial monolayer while removing non-adherent NB4 cells. The number of adhered NB4 cells was determined by manually counting cells from light microscope images (3–5/sample) at a magnification of 4 ×.

### Protein collection and western blot

HAAECs were washed with cold PBS and lysed in situ in cold RIPA lysis buffer (ThermoFisher, 89900) with 2% Halt™ protease and phosphatase inhibitor cocktail (ThermoFisher, 78440). Samples were vortexed and kept on ice before centrifuging at 13,000 RPM for 10 min. Protein quantification was performed through colorimetric assays and protein quantities were normalized between samples. Loading samples were prepared with Bolt LDS sample buffer and reducing agent (ThermoFisher, B0007, B0009). Gel electrophoresis were performed using the Mini Blot Gel tank and pre-cast Bolt 4–12% Bis–Tris Plus gels loaded with 20 μg of protein. Protein transfer was carried out using the Mini Blot module and 0.45 μm nitrocellulose membranes following manufacturer instructions. Following the transfer, membranes were blocked with 5% bovine serum albumin (BSA, BioShop, ALB001) in 0.1% Tween-20 TBS (TBST) for 1 h. Primary antibodies were diluted in blocking buffer and incubated at 4 °C overnight on a shaking plate. Antibodies consisted of eNOS (1:500, BD Biosciences, 610297), phospho-eNOS Ser1177 (1:500, Abcam, ab184154), ICAM-1 (1:5000, Abcam, ab109361), GAPDH (1:10,000, ThermoFisher, AM4300) and TXNIP (1:1000, Abcam, ab188865). After 3 washes in TBST, the membranes were incubated with horseradish peroxidase secondary antibodies (1:40,000, ThermoFisher) diluted in blocking buffer for 1 h at room temperature. Membranes were washed 3 times in TBST and signal detection was achieved with SuperSignal™ West Pico PLUS Chemiluminescent Substrate and UVP Biospectrum 810 MultiSpectral Imaging System. Proteins were quantified by densitometry using ImageJ and normalized to the loading control (GAPDH).

### Transcriptome analysis

The transcriptome profiles of HAAECs under two sets of conditions were studied. Cells were statically cultured with or without EMPA for 6 h or cultured under similar conditions to the adhesion assay (24 h shear culture, TNF-α stimulation, CTL (Static), CTL, sDEG and sDEG-EMPA). Following treatment, cells were lysed in situ and RNA isolation was performed using the RNeasy Plus Micro kit (QIAGEN) according to manufacturer instructions. Sample RNA integrity was confirmed with the RIN using a BioAnalyzer (Agilent). The genomic transcriptome profile of each sample was determined with a Human Clariom S Array and analyzed using the Transcriptome Analysis Console software (SST-RMA method, Applied Biosystems, v4.0.2). Gene lists were generated by filtering minimal threshold expression and fold changes (≤ − 1.5 or ≥ 1.5). The open-source pathway database REACTOME was used to analyze the gene lists and to identify potential pathways based on the number of matched entities^61^.

### Reactive oxygen species measurement

Reactive oxygen species (ROS) production was measured using the 2′,7′-dichlorofluorescin diacetate (DCFDA) assay kit (Abcam, ab113851) according to the manufacturer instructions. Briefly, ECs were cultured in 96-well plates at a density of 30,000 cells/cm^2^ and stained with 10 μM H2DCFDA for 30 min prior to treatments. Tert-Butyl hydroperoxide (TBHP) at 50 μM was used as positive control, with or without 50 μM EMPA for 6 h. Pretreatment with EMPA (preEMPA + TBHP) was also tested by incubating with EMPA for 18 h prior to the assay. TNF-α (10 ng/mL) and sDEG over 24 h were also tested. After incubation, ROS production was measured by fluorescence unit on a Berthold fluorescence microplate reader with the FITC filter set. Measurements were normalized to the control sample i.e. H2DCFDA stained cells with no TBHP or EMPA treatment.

### Statistical analysis

Statistical analysis was performed using GraphPad Prism™ software. Results are presented as the mean values ± standard deviation of 3 independent experiments. Each experiment included 2–6 replicates for each condition and the mean was used in the analysis. When applicable, the normality of the data was confirmed by D’Agostino and Pearson test. Otherwise, the data was assumed to be normally distributed. Mean values were compared using *t* tests, one-way or two-way analysis of variance (ANOVA) followed by Bonferroni post-test with a 95% confidence interval. Statistical analysis of the transcriptome data was carried out through the Transcriptome Analysis Console software using default settings with eBayes analysis. p-values less than 0.05 were considered significant.

## Supplementary Information

Supplementary Information.

Supplementary Table S1.

## Data Availability

The datasets generated for this study are available on request to the corresponding author.
