# Supplementary material for: Empagliflozin mitigates endothelial inflammation and attenuates endoplasmic reticulum stress signaling caused by sustained glycocalyx disruption
Source: Sci Rep. 2022 Jul 25;12:12681. doi: 10.1038/s41598-022-16763-6 (PMC9314417; doi:10.1038/s41598-022-16763-6)
Supplement: Supplementary file 1 — Supplementary Information. [file 41598_2022_16763_MOESM1_ESM.pdf]

Supplementary information

Empagliflozin mitigates endothelial inflammation and attenuates endoplasmic reticulum stress signaling caused by sustained glycocalyx disruption

Marc-Antoine Campeau[1], Richard L. Leask[1,2*]

[1]Department of Chemical Engineering, McGill University, Montreal, QC, Canada

[2]McGill University Health Centre, Montreal, QC, Canada

*Corresponding Author

richard.leask@mcgill.ca

Keywords: Sodium/glucose cotransporter 2 (SGLT2) inhibitor, empagliflozin (EMPA), endothelial cell dysfunction, type 2 diabetes, endoplasmic reticulum stress (ER stress), glycocalyx, inflammation, oxidative stress

EMPA does not restore HS integrity under sustained degradation

HS integrity and the impact of EMPA under shear conditions were studied through HS immunostaining. The initial condition of degradation has shown to reduce the mean HS intensity by approximately 85% compared to control (Fig. S1, DEG T0 vs CTL T0 * p<0.001). The mean HS intensities were similar between the EMPA-treated cells and the control after 24h (EMPA vs CTL). Cells under sustained degradation showed minimal regain in intensity indicating continued degradation of HS over the 24h period of culture (sDEG & sDEG-EMPA vs CTL # p<0.001; vs CTL T0 * p<0.001) with no effect of EMPA. Taken together, these results suggest that treatment with EMPA for 24h does not enhance HS on EC surface or prevent the effect of sustained degradation.

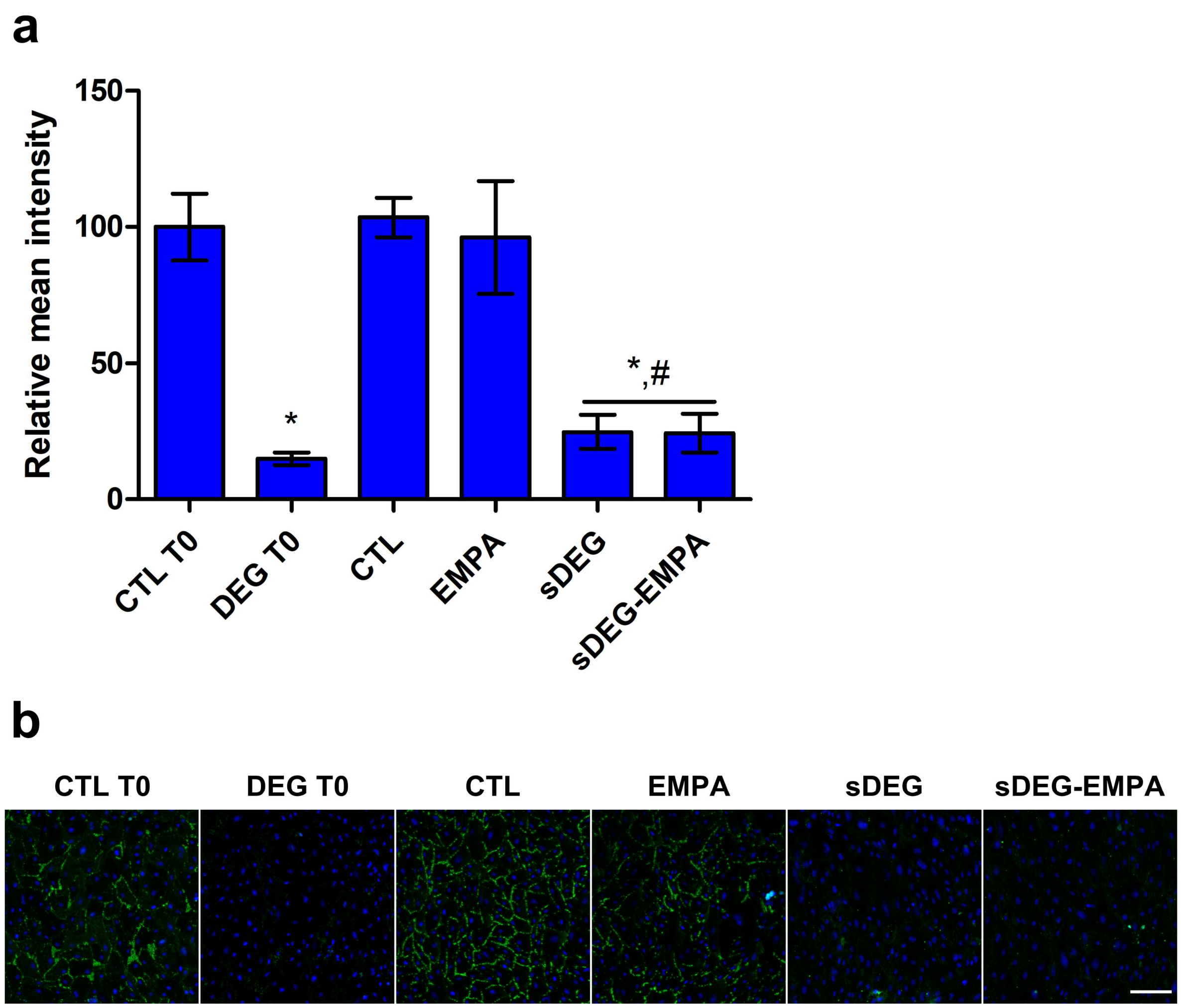

Supplementary Figure S1. Relative mean intensity of HS immunostaining images to assess HS integrity. (**a**) Relative intensity of HS at initial conditions (T0) and 24h of shear culture. Initial condition following degradation (DEG T0) reduced HS intensity by approximatively 85% compared to control (DEG T0 vs CTL T0 * p<0.001). Sustained degradation maintained HS degraded over 24h compared to control (sDEG & sDEG-EMPA vs CTL # p<0.001; vs CTL T0 * p<0.001). (**b**) Representative HS immunostaining images (scale bar = 200 μm).

EMPA does not restore cell elongation in response to shear under sustained HS degradation

In accordance with previous results, EMPA caused cell elongation (i.e. reduction in shape index (SI)) in static culture (Fig. S2, EMPA vs CTL * p<0.001). The EMPA-induced elongation also persists under sustained degradation in static culture (sDEG-EMPA vs sDEG # p<0.001; vs CTL * p<0.001). Conversely, the effect of EMPA on shear-induced elongation was absent under degradation with impaired cell elongation as shown by the increased SI (sDEG & sDEG-EMPA vs CTL * p<0.001), suggesting that EMPA does not alleviate EC dysfunction caused by the loss of normal HS mechanotransduction.

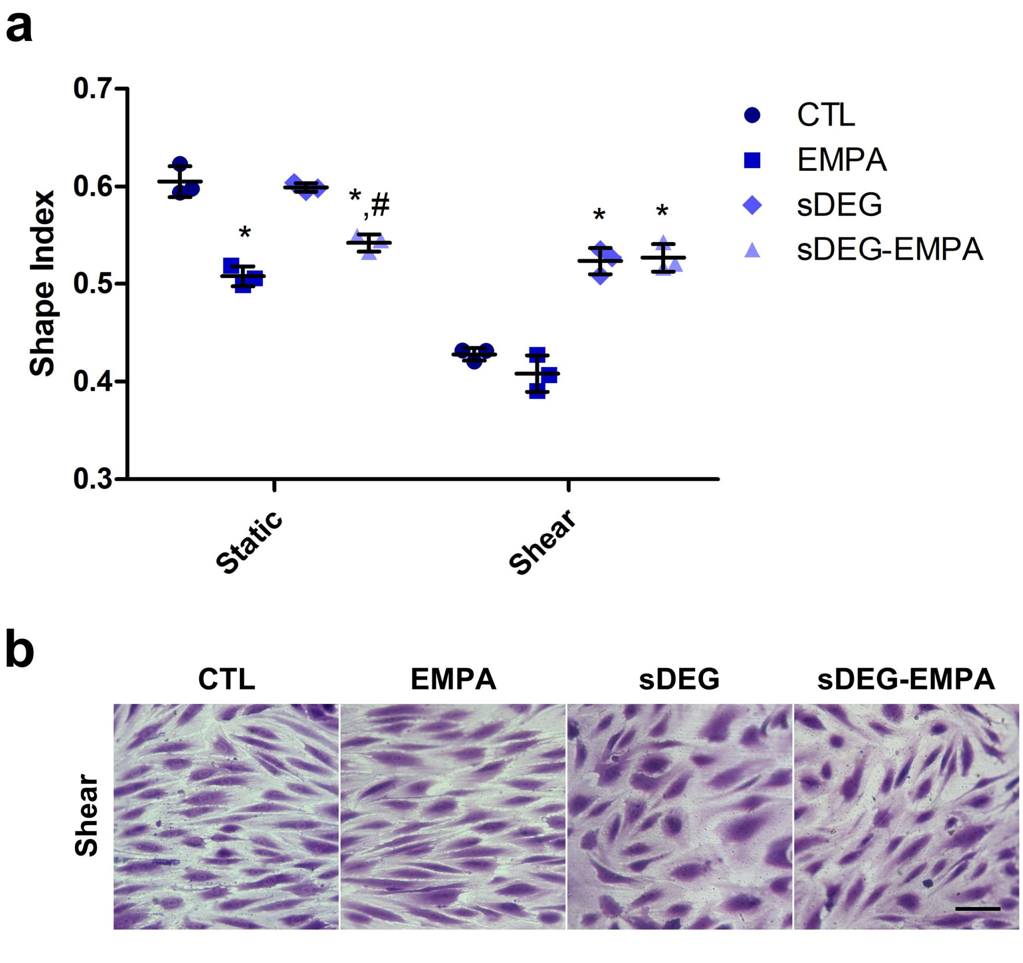

Supplementary Figure S2. HAAEC cell elongation under sustained HS degradation with or without EMPA treatment in static and shear conditions for 24h. (**a**) SI measurements. EMPA induced reduction in SI in static culture (EMPA vs CTL * p<0.001, sDEG-EMPA vs sDEG # p<0.001; vs CTL * p<0.001). Sustained degradation under shear caused an increase in SI compared to control vs CTL (sDEG & sDEG-EMPA vs CTL * p<0.001) with no effect of EMPA. (**b**) Representative images of shear culture conditions (scale bar = 200 μm).

EMPA does not directly reduce ROS production caused by TBHP

We sought to determine if EMPA has a direct protective effect against oxidative stress. Treatment with EMPA for 6h did not reduce the baseline ROS production level (Fig S3, EMPA vs CTL). While incubation with TBHP resulted in a significant increase in measured ROS after 6h (TBHP vs CTL * $p<0.01$), simultaneous treatment with EMPA for 6h or pretreatment with EMPA for 18h (preEMPA+TBHP) failed to limit the increase (TBHP+EMPA vs CTL * $p<0.01$, preEMPA+TBHP vs CTL # $p<0.05$). Adhesion assay conditions (TNF-α stimulation and sustained HS degradation) did not show significant ROS production compared to control in static culture (data not shown).

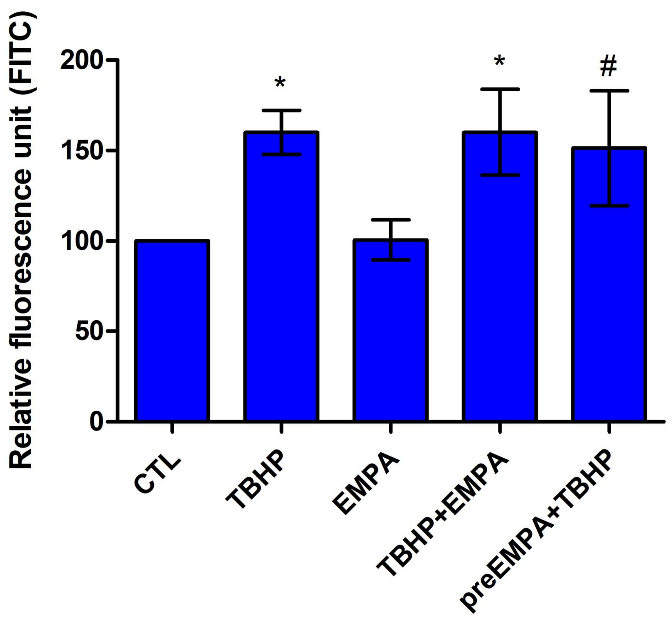

Supplementary Figure S3. Relative ROS production measurements using the H2DCFDA ROS assay kit. TBHP used as a positive control resulted in increased ROS production after 6h compared to CTL (TBHP vs CTL * $p<0.01$). Simultaneous incubation (TBHP+EMPA) or preincubation (preEMPA+TBHP) with EMPA did not reduce the TBHP-induced increase in ROS production (TBHP+EMPA vs CTL * $p<0.01$, preEMPA+TBHP vs CTL # $p<0.05$).

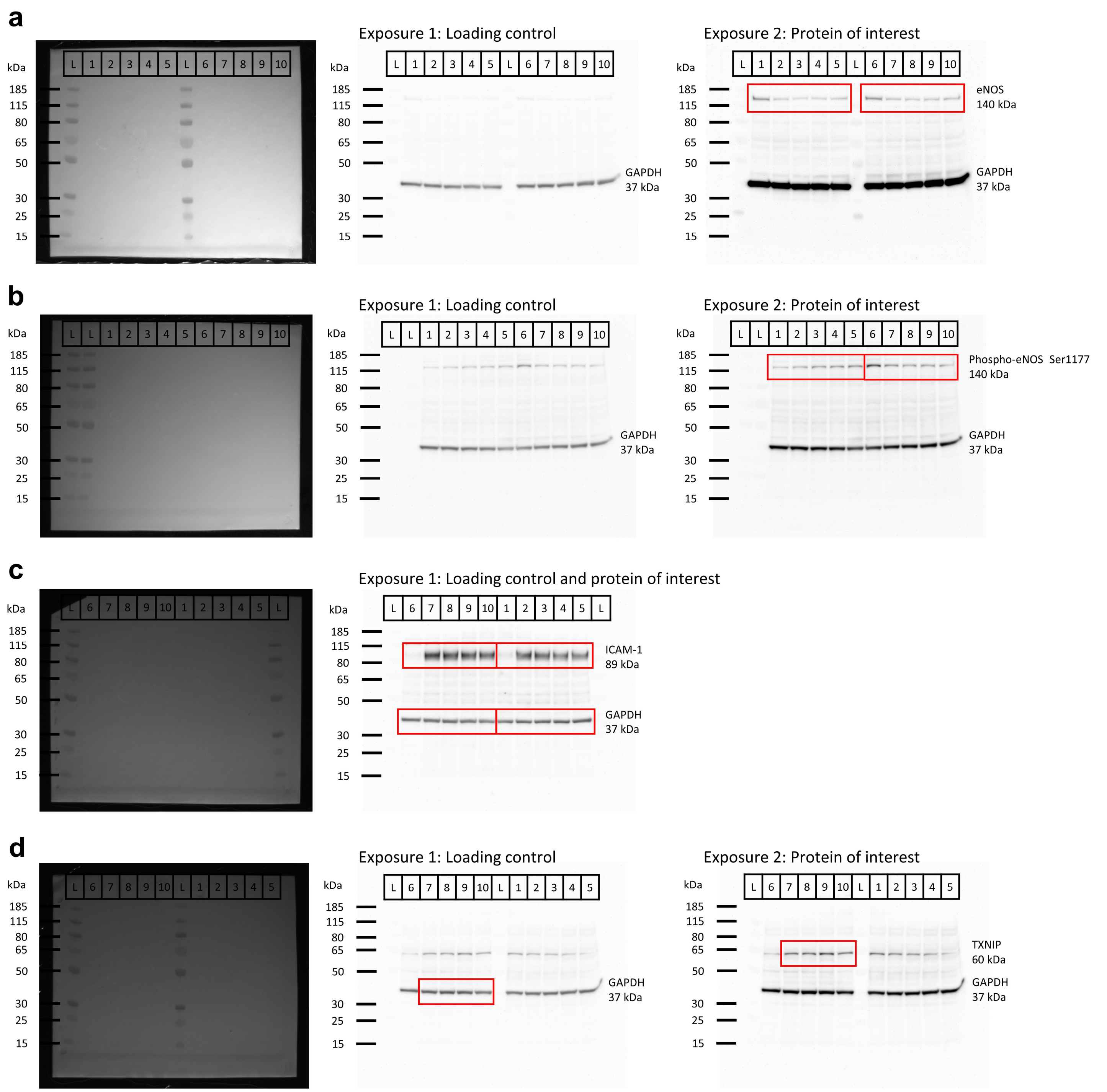

Supplementary Figure S4. Whole Western blots from Figure 2e and 4d. Full-length blots of (**a**) eNOS, (**b**) phospho-eNOS Ser1177, (**c**) ICAM-1, (**d**) TXNIP and the loading control GAPDH. Blots are presented as unprocessed images obtained from the UVP Biospectrum 810 MultiSpectral Imaging System. Bright field images and multiple exposures of the same blot, when applicable, are presented in columns. The cropped areas used in Figure 2e and 4d are indicated by boxes. Samples included (1) CTL (TNF-α –) Static, (2) CTL (TNF-α +) Static, (3) EMPA (TNF-α +) Static, (4) sDEG (TNF-α +) Static, (5) sDEG-EMPA (TNF-α +) Static, (6) CTL (TNF-α –) Shear, (7) CTL (TNF-α +) Shear, (8) EMPA (TNF-α +) Shear, (9) sDEG (TNF-α +) Shear, (10) sDEG-EMPA (TNF-α +) Shear, (L) Protein ladder.
